# Folk-Lore in Medicine

**Published:** 1921-12

**Authors:** 


### FOLK-LORE IN MEDICINE.

### THE PASSING OF THE LEECH.

THE trade in leeches is one of the dying trades. The leech, which fifty years ago reigned in solitary state, to-day has almost disappeared, dethroned by the simple cupping-glass. Before 1870 a dozen or so merchants in Paris made their living out of this pharmaceutical "game." Each of them sold between three and four hundred thousand leeches a month, at 250 francs per thousand. To-day Paris possesses only one firm interested in this form of commerce, and its trade has sunk to a hundred and thirty thousand per month, purchased at a rate of 60 francs per thousand. The "Assistance Publique," which in 1849 bought leeches to the value of 80,000 francs, now spends less than 200 francs in such purchases. Were it not for the export trade it would be impossible for even one man to make a living out of leeches. Luckily, the United States still remain faithful to old customs and are by far the best customers of the "hirudiculturist." Formerly leech farms existed at Bordeaux, where the young "boarders" were bred and brought up at the expense of old horses, which were made five or six times a month to paddle in the artificial ponds containing the leeches. At the present day the leech is not artificially bred, but is fished for in Croatia, Dalmatia, and Turkey. It is exported in baskets like oysters or in turf-lined cases. On its arrival in France it is placed in clay-lined pigeon-holes in a cellar, where, fasting, it awaits the moment when it is to take its revenge on some patient. Though the "Assistance Publique" has renounced its services, the naval authorities still continue to make use of it, and a good story is told concerning a bottle of leeches purchased in 1907. Each animal on its arrival had to be given a doctor's certificate stating that it had turned up in good condition. It happened, however, that five of them were made to die a glorious death before such certificates could be obtained. It needed two years of inquiries, references to commissions, and reports to Ministers to justify their disappearance "from force of circumstances."

### THE MOST ANCIENT PHYSICIAN IN THE WORLD.

THE first physician whose skill has been duly attested is the Egyptian I-em-Hetep (The Bearer of Peace) who lived under the King Tehser of the Third Dynasty, that is to say, probably about 4500 B.C. During his lifetime he enjoyed considerable fame, for he was buried with royal honours by the side of the royal tomb in the pyramid of Sakkarah, near Memphis. According to an old tradition he was surnamed "Master of the Mysteries and Cyphers," no doubt on account of the extraordinary number of drugs in use among the ancient Egyptians. His memory was venerated by the people for more than four thousand years, and his name was given to many hospitals. Later, in the reign of the Ptolemies, his name was identified with that of the Greek god of medicine, Aesculapius, who lived three thousand years after his illustrious predecessor. Three centuries after I-em-Hetep, the King Atoti wrote a treatise on anatomy, and a few centuries later there lived another celebrated surgeon, in whose tomb, near Sakkarah, were discovered very interesting drawings representing various surgical operations. From the celebrated Ebers Papyrus, which contains many curious details on the art of healing among the ancient Egyptians, it must be supposed that the representatives of medicine were already in great repute among this people six thousand years before Christ.

### SOME BRETON CHARMS.

THE habit of carrying amulets against various diseases is an exceedingly common one at the present day in the rural districts of Brittany, especially among the children. The amulets comprise sachets, medals, necklets, and neck-cords. In addition to those used therapeutically there are others used for religious purposes. At times the same amulet has both uses, and, in fact, it is often difficult to differentiate between them. Usually the charms are carried directly applied to the wearer's skin, the idea being that direct contact intensifies their action. They are not indifferently applied to any portion of the body, but each has a definite place assigned to it, such as the neck, the thorax, the precordial region or the abdomen. *Amulet against the thrush.*—This possesses an essentially medical character. Thrush, or "chancre," as the Vendéan peasants call it, is of relatively frequent occurrence among young children. It must be cured without medical intervention. On the chest of the small patient is placed a special amulet in some way or other considered specific against the affection. It is held in place by two small cords attached round the neck. The charm itself consists of a small linen or flannel bag about 5 or 6 centimetres square sewn together by hand. This small bag is left in place until the cure is complete, which should take place in four or five days. The charms can be used over and over again, and to that end are always preserved after use. They are usually sold by people in the habit of making them, and each district or village possesses some old woman whose "speciality" they are. Sometimes, however, in urgent cases, other persons knowing the secret of their composition supply them. The contents of the bag are as follows: Five tips of broom, five of bramble, five of black currant, and a dried worm, the whole being wrapped in paper. *Amulet against Worms.*—This consists of a collar made of cloves of garlic threaded together lengthwise by means of an ordinary string. This charm acts both as a preventive and as a cure. It is applied at the time when the attacks of worms come on. There exists also another amulet which prevents convulsions due to worms. This is worn permanently. It can be bought commercially at the draper's, consisting merely of an ordinary collar of glass beads.

